# A novel mutation of *AFG3L2* might cause dominant optic atrophy in patients with mild intellectual disability

**DOI:** 10.3389/fgene.2015.00311

**Published:** 2015-10-19

**Authors:** Majida Charif, Agathe Roubertie, Sara Salime, Sonia Mamouni, Cyril Goizet, Christian P. Hamel, Guy Lenaers

**Affiliations:** ^1^Institut des Neurosciences de Montpellier, U1051 de l’INSERM, Université de MontpellierMontpellier, France; ^2^PREMMi, CNRS UMR 6214 – INSERM U1083, Département de Biochimie et Génétique, Université d’Angers, CHU d’AngersAngers, France; ^3^CHRU Montpellier, Service de Neuro-pédiatrie, Hôpital Gui de ChauliacMontpellier, France; ^4^CHRU Montpellier, Centre de Référence pour les Maladies Sensorielles Génétiques, Hôpital Gui de ChauliacMontpellier, France; ^5^CHU Bordeaux, Service de Génétique Médicale and Université de Bordeaux, Laboratoire Maladies Rares, Génétique et Métabolisme (MRGM)Bordeaux, France

**Keywords:** optic nerve, dominant mutation, *AFG3L2*, *OPA1*, retinal ganglion neurons

## Abstract

Dominant optic neuropathies causing fiber loss in the optic nerve are among the most frequent inherited mitochondrial diseases. In most genetically resolved cases, the disease is associated to a mutation in *OPA1*, which encodes an inner mitochondrial dynamin involved in network fusion, cristae structure and mitochondrial genome maintenance. OPA1 cleavage is regulated by two *m-*AAA proteases, SPG7 and AFG3L2, which are, respectively involved in Spastic Paraplegia 7 and Spino-Cerebellar Ataxia 28. Here, we identified a novel mutation c.1402C>T in *AFG3L2*, modifying the arginine 468 in cysteine in an evolutionary highly conserved arginine-finger motif, in a family with optic atrophy and mild intellectual disability. Ophthalmic examinations disclosed a loss of retinal nerve fibers on the temporal and nasal sides of the optic disk and a red–green dyschromatopsia. Thus, our results suggest that neuro-ophthalmological symptom as optic atrophy might be associated with *AFG3L2* mutations, and should prompt the screening of this gene in patients with isolated and syndromic inherited optic neuropathies.

## Introduction

Dominant optic atrophy (DOA) is a neurodegenerative disease that primarily targets the retinal ganglion cell (RGC), representing an important cause of early onset blindness. In most patients, the clinical presentation is restricted to visual impairment, but in about 20% of them, other symptoms are associated such as deafness, muscle weakness, and peripheral neuropathy (Yu-Wai-Man et al., 2010; Lenaers et al., 2012; Maresca et al., 2013). DOA is caused by mutations mostly in *OPA1* (Alexander et al., 2000; Delettre et al., 2000; Ferre et al., 2015) and rarely in *OPA3* (Reynier et al., 2004) or *SPG7* (Klebe et al., 2012), all encoding mitochondrial inner membrane proteins. In some cases, DOA associated to deafness is caused by *WFS1* mutations (Rendtorff et al., 2011), encoding an endoplasmic reticulum protein, regulating calcium homeostasis and consequently mitochondrial physiology. Nevertheless, the systematic screening by Sanger sequencing of exonic sequences in *OPA1*, *OPA3*, and *WFS1* genes in our DOA cohort only allowed for about 50% of genetic diagnoses. We further excluded *OPA1* deletion/duplication in all patients, by multiplex ligation-dependent probe amplification (SALSA MLPA KIT P229-B1 OPA1 – MRC-Holland), except one, who presented a full *OPA1* deletion.

**FIGURE 1:**
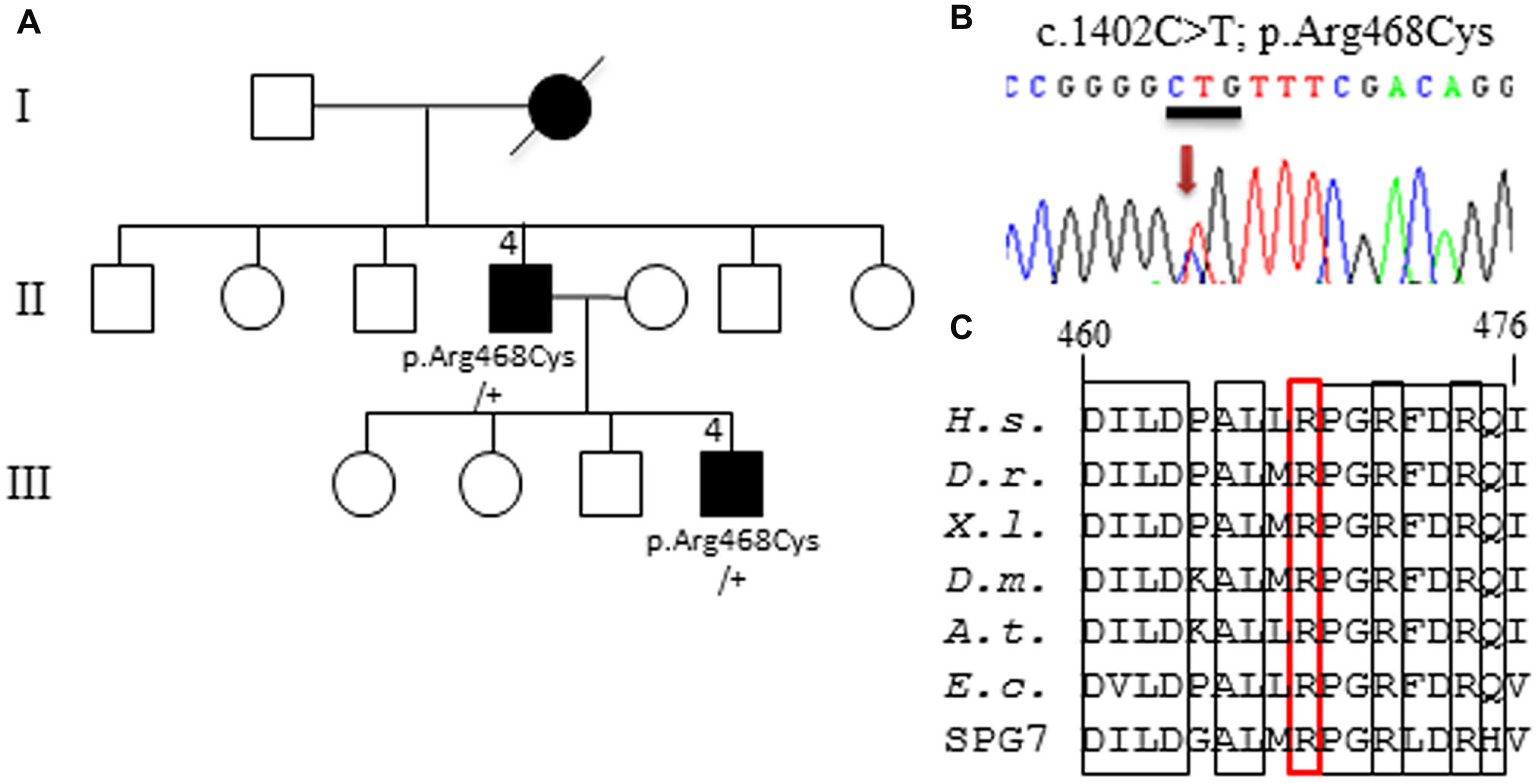
**Identification of *AFG3L2* mutation. (A)** Pedigree showing the family with the affected members in black, and the segregation of the p.Arg468Cys amino-acid change in the two affected patients, knowing that DNA sample was not available for the other family members. **(B)** Electrophoregram presenting the *AFG3L2* c.1402C>T heterozygous mutation. **(C)** Alignment of AFG3L2 orthologs (amino-acids 460 to 476) showing the evolutionary conserved positions around the Arginine 468, which is squared in red in the triple arginine motif (bold rectangles). (*H.s. Homo sapiens; D.r. Danio rerio; X.l. Xenopus laevis; D.m. Drosophila melanogaster; A.t. Arabidopsis thaliana; E.c. Escherichia coli* FtsH protein*;* SPG7: human paralog of AFG3L2).

Thus, to deepen DOA genetic diagnosis, we performed whole exome sequencing (WES) on the proband from 12 unresolved DOA families. We report here the results obtained for one family, presenting an Optic Atrophy and mild intellectual disability (**Figure 1A**). Amongst the 60,000 variants identified by WES on the index patient III.4, no mutation was found in *OPA1*, *OPA3*, and *WFS1* genes, that were covered by a minimal sequencing depth of 50x, 44x, and 40x, respectively. We also carefully excluded mutations in eleven genes (*AIFM1*, *ATP1A3*, *c12orf65*, *CISD2*, *FXN*, *MTPAP*, *PRPS1*, *SFXN4*, *SPG7*, *TIMM8*, and *TSFM*), for which mutations might cause clinical presentations related to our patients (e.g., Mohr-Tranebjaerg and CAPOS syndromes). Thus, we progressively filtered WES data. After excluding intronic, 5′UTR, 3′UTR and synonymous variants, we obtained 14,241 exonic and splicing variants. A total of 9,776 variants were selected with a number of call higher than 4 using the GATK, VarScan, SNVer, Lofreq, Platypus softwares. We discarded common variants reported in NHLBI Exome Sequencing Project, Exome Variant Server, NCBI, the 1000 genomes and ExAC ver0.2, with a frequency greater than 1%, leading to 145 left variants. Then 30 deleterious SNPs predicted by SIFT, PolyPhen-2, and Mutation Taster softwares were retained (see Supplementary Table S1). Finally, these variants were filtered for heterozygous mutations in gene encoding mitochondrial protein (MitoCarta), as to date all DOA genes are involved in mitochondrial physiology. We only found the c.1402C>T heterozygous mutation in *AFG3L2* exon 11 (**Figure 1B**) that was never reported before in any database (see above for a list). It modifies an evolutionary highly conserved arginine 468 in cysteine (p.Arg468Cys) within an arginine-finger motif (RPGRxxR) that is found in the AAA domain of all AFG3L2 orthologs and SPG7 paralogs. The arginine 468 is also conserved in the prokaryote FtsH from *E. coli* (**Figure 1C**). *In silico* prediction of the p.Arg468Cys pathogenicity by Polyphen2, SIFT, Mutation taster, imutant, Pmut, MetaSVM, MetaLR, FathMM, and PhD-SNP programs gave the highest scores, supporting the pathogenicity of this mutation.

### Patient II.4

**FIGURE 2:**
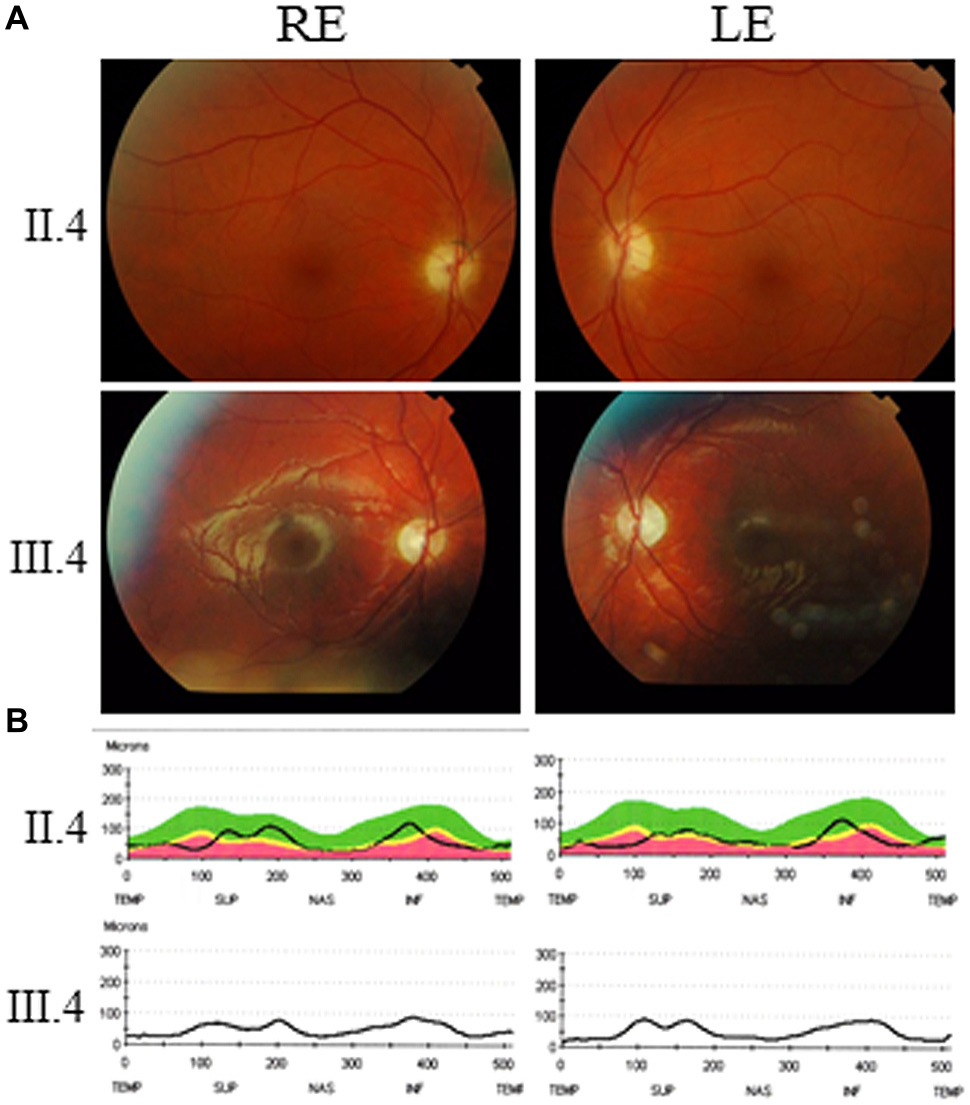
**Ophthalmological exploration of *AFG3L2* patients. (A)** Eye fundus (RE: right eye; LE: left eye) from patient II.4 (top) and patient III.4 (bottom) disclose temporal pallor of the optic disks. **(B)** Optical Coherence Tomography scanning and measurement of the retinal nerve fiber layers of the optic disks evidenced a drastic thickness reduction in the temporal and nasal quadrants of patient II.4 (top) and III.4 (bottom).

This 48-year-old male complained of visual difficulties since 12 years of age including decrease in visual acuity, photophobia, and color vision impairment with very slow progression of visual loss. He otherwise had normal development without neuromuscular or other sensory involvement, but he had mild intellectual disability. His best corrected visual acuity was decreased to 0.3 and 0.4 in the right and left eyes, respectively. Fundus examination revealed bilateral moderate optic disk pallor (**Figure 2A**, top), and optical coherence tomography (OCT) disclosed a moderate overall decrease in the thickness of the retinal nerve fiber layer (**Figure 2B**, top). Color vision testing showed a red/green axis of dyschromatopsia and visual field indicated a decrease in light sensitivity without scotoma (not shown). He refused further neurological examination and MRI scanning.

### Patient III.4

His son was born after a normal pregnancy and delivery was uneventful. Motor development was normal, he managed to walk unaided at 14 months. Learning difficulties were noticed in primary school. Cognitive evaluation disclosed a moderate intellectual disability (Wechsler Intelligence Scale III: Total Intellectual Quotient 47) and the patient benefited of a special educational program from the age of 8, and was admitted in an institution for patients with mental impairment from the age of 14. This patient had no clinical complaint especially no unsteadiness, no clumsiness, no cramping, no fatigability. Clinical examination was performed at the age of 18 and disclosed a discrete Kyphosis, while motor strength, sensory evaluation, osteotendinous reflexes and cranial motor nerve functions were normal. Oculomotor movements were normal. The patient did not exhibit any sign of stato-kinetic ataxia; his family never noticed any jerk, and clinical examination did not disclose myoclonias. Audiological assessment including tonal and vocal audiometry was normal.

**FIGURE 3:**
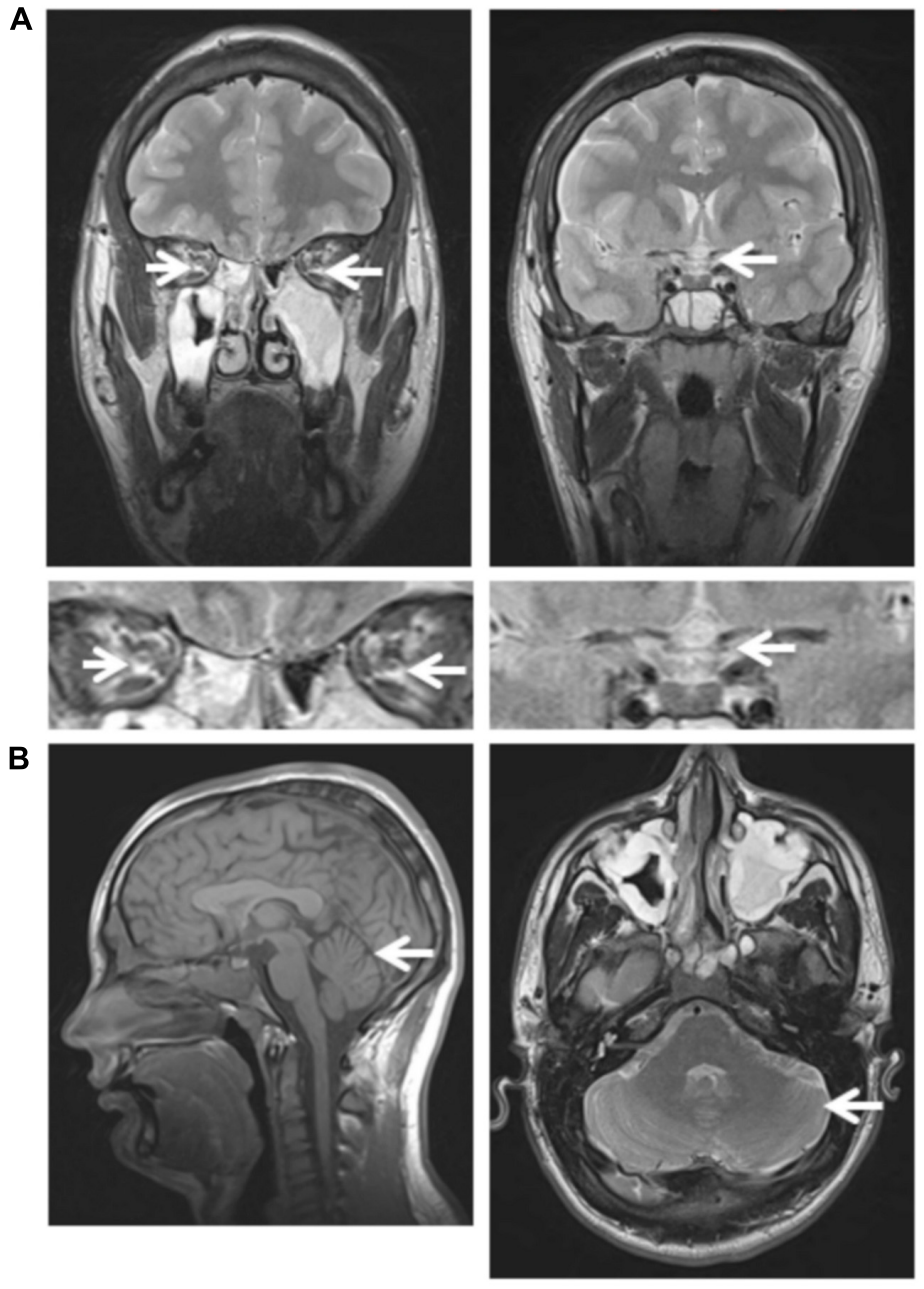
**Brain MRI of patient III.4 at 18 years of age. (A)** Left: Frontal section, T2 sequence, showing atrophy of the optic nerve (arrow) and Right: reduced size of the chiasma (arrow). Magnification of the optic track (Left) and chiasma (Right) regions are shown underneath the full picture. **(B)** Left: Sagittal section, T1 sequence, showing a normal aspect of the brain, especially of the vermis (arrow); Right: axial section, T2 sequence, showing a normal aspect of the posterior fossa, especially the cerebellar hemisphere (arrow).

Ophthalmological investigations disclosed a best corrected visual acuity of 0.5 on both eyes, with difficulties to fixate his gaze. Fundus examination revealed bilateral severe optic disk pallor (**Figure 2A**, bottom) and OCT examination disclosed a marked overall decrease in the thickness of the retinal nerve fiber layer (**Figure 2B**, bottom). His brain MRI disclosed reduced size of the optic tract (**Figure 3A**, left) and atrophy of the optic chiasma (**Figure 3A**, right), without any sign of cerebellar atrophy (**Figure 3B**, left and right). MRI spectroscopy was normal.

Both patients denied to do further examination, therefore skin biopsy and brain MRI of the father were not available. The other family members were not compliant with clinical and genetic analysis.

## Background

The AFG3L2 and SPG7 *m-*AAA proteases have crucial multiple functions in the homeostasis of mitochondrial protein synthesis, assembly and degradation. On the one hand, homo and hetero-polymers of these proteases have broad functional targets, as for example proteins involved in the assembly of ribosomes and respiratory chain complexes, the degradation of misfolded proteins and the maintenance of mitochondrial nucleoids. On the other hand, they have also highly specific targets as the proteolytic cleavage of MRLP32 and OPA1 (Martinelli and Rugarli, 2010). The latter cleavage in OPA1 amino-terminal region is required for the physiological equilibrium between pro-fusion and pro-fission isoforms of this large GTPase (Ehses et al., 2009). In this respect, AFG3L2 down-regulation affects respiratory capacity and calcium uptake, and induces mitochondrial network fragmentation (Maltecca et al., 2012), a cellular phenotype that we also found in cell lines with altered OPA1 expression (Olichon et al., 2007; Chevrollier et al., 2008; Dayanithi et al., 2010). The possible consequences of *AFG3L2* mutation might be related to the pathophysiology of DOA, a disease which is caused by OPA1 haplo-insufficiency or eventually by a dominant negative process (Olichon et al., 2007). Thus, the p.Arg468Cys mutation might affect AFG3L2 specific interaction with OPA1 long isoform and consequently its processing, leading to a bias in OPA1 physiological maturation and pro-fusion activity.

To date, three genes have been identified as responsible for non-syndromic DOA: *OPA1*, *OPA3*, and *SPG7* (Alexander et al., 2000; Delettre et al., 2000; Reynier et al., 2004; Klebe et al., 2012), encoding proteins embedded in the inner mitochondrial membrane (Olichon et al., 2002; Atorino et al., 2003; Grau et al., 2013), and involved directly or indirectly in mitochondrial fusion and fission (Olichon et al., 2003; Ishihara et al., 2006; Ryu et al., 2010). *OPA1* mutations are by far the most frequently found in patients with non-syndromic DOA, and to date more than 250 mutations have been identified in this gene (Ferre et al., 2015). Three and one dominant mutations were respectively identified in *OPA3* and *SPG7* genes. In virtually all cases, patients show optic disk atrophy with a predominant loss of the temporal retinal nerve fiber bundle revealed by OCT examination (Klebe et al., 2012; Lenaers et al., 2012). Nevertheless, it should be stressed that DOA is frequently associated to secondary symptoms, most often neurosensory deafness (Amati-Bonneau et al., 2005), but also external ophthalmoplegia, myopathy, peripheral neuropathy, ataxia, and spastic paraplegia for *OPA1* patients (Yu-Wai-Man et al., 2010), and cataract, postural tremor, extrapyramidal signs, ataxia, areflexia, and neurosensory deafness for *OPA3* patients (Reynier et al., 2004; Ayrignac et al., 2012; Grau et al., 2013). Concerning *SPG7*, most recessive patients with primary spastic paraplegia also present optic atrophy, whereas in the single family reported with a dominant *SPG7* mutation, patients had isolated optic atrophy (Klebe et al., 2012).

Importantly, combined optic nerve atrophy and encephalopathy were not previously reported in families with dominant inheritance, whereas this is encountered in syndromic recessive forms of optic atrophy (Metodiev et al., 2014). Thus the clinical presentation that we report here is somehow particular in associating DOA to mild intellectual disability.

**FIGURE 4:**
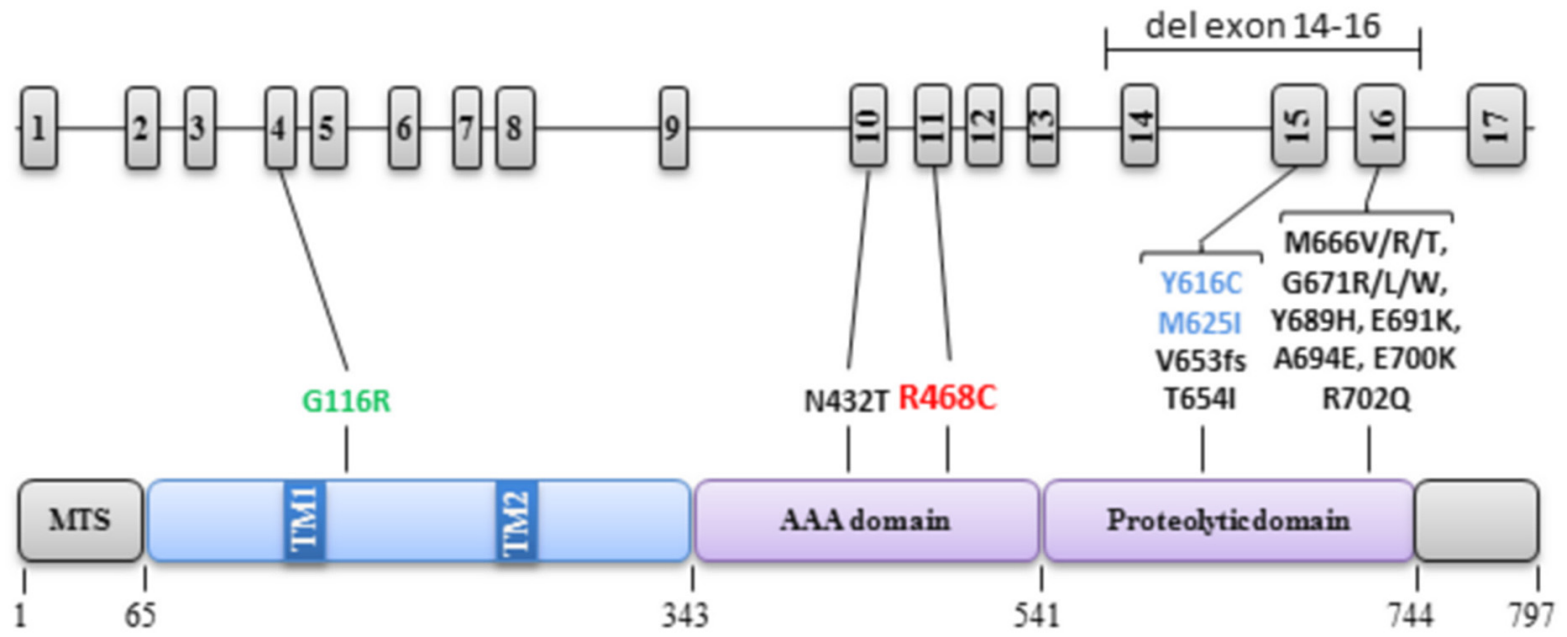
**Localization of *AFG3L2* mutations in the gene and protein**. The structures of *AFG3L2* gene (17 exons) and the protein (domains and amino-acid positions) are described on the top and bottom of the figure, respectively, with all the pathogenic mutations and associated clinical presentation reported to date. In black: SCA28, in blue: SPAX5, in green: Myoclony and pyramidal signs, in red: present case.

Mutations in *AFG3L2* are responsible for the SCA28 and SPAX5 forms of spinocerebellar ataxia (Di Bella et al., 2010; Pierson et al., 2011). In SCA28, most cases are caused by heterozygous mutations in exon 15 and 16, with a single exception (c.1295A>C, p.N432T) in exon 10 (Cagnoli et al., 2010; Di Bella et al., 2010), whereas in SPAX5, two families were reported with homozygous mutations in exon 15 (c.1847A>G, p.Y616C and c.1875G>A, p.M625I; Pierson et al., 2011; Muona et al., 2015), (**Figure 4**). Functional studies of the encoded *m-*AAA protease with mutation in exons 15 or 16 revealed an abnormal C-terminus M41 peptidase domain compromising the stability and proteolytic activity of the *m-*AAA complex, possibly through a dominant negative process. By contrast, the exon 10 mutation modifies the structure of the pore channel entry, in which substrates are pulled to be degraded (Di Bella et al., 2010). In addition, two *AFG3L2* gene deletions have been reported in *SCA28* patients, one of the entire gene (Myers et al., 2014), and one including exon 14 to exon 16 (Smets et al., 2014), as well as one frame shift mutation in exon 15, deleting the last 144 amino acids (Musova et al., 2014), (**Figure 4**). Thus AFG3L2 haplo-insufficiency is one of the possible causes of SCA28. Very recently, a novel c.346A>G, p.G116R mutation in the N-terminal domain of AFG3L2 protein was reported in a patient presenting spontaneous myoclonus at proximal limb associated to pyramidal signs. As for the cases we report here, this young patient did not present cerebellar ataxia, oculomotor abnormalities or sensory deficits (Mancini et al., 2015), although we cannot exclude that they will develop these symptoms later in life.

In both SCA28 and SPAX5 presentations, the patients have a typical cerebellar syndrome with ataxia, dysarthria, and oculomotor apraxia, variously associated with pyramidal and muscular signs, eventually including ptosis and external ophthalmoplegia (Cagnoli et al., 2010; Di Bella et al., 2010; Pierson et al., 2011). In none of them, optic atrophy, cognitive deficit or intellectual disability has ever been reported. Yet, optic atrophy is found associated with spastic paraplegia linked to recessive mutations in *SPG7*, the paralog of *AFG3L2* encoding another component of the *m-*AAA protease involved in OPA1 proteolytic maturation (Casari et al., 1998; Arnoldi et al., 2008). In addition, genetic analysis of a large family with isolated optic atrophy disclosed the first dominant *SPG7* mutation, c.1232A>C, p.D411A, affecting the AAA domain (Klebe et al., 2012), 56 amino-acids upstream of the conserved RPGR motif, in which we found the AFG3L2 mutation.

## Discussion

Both patients described in this study, who harbored a novel *AFG3L2* mutation, had typical signs of hereditary optic atrophy, without abnormal gait or signs of cerebellar impairment. Indeed, they complained of bilateral, symmetrical visual loss starting in infancy and they showed optic disk pallor with thinned retinal nerve fiber layer. Dyschromatopsia was also present in one of the patient. This is in contrast with the SCA28 and SPAX5 presentations in which optic atrophy has never been reported. Yet, it is possible that asymptomatic optic atrophy exists in SCA28 and SPAX5 patients since decreased visual acuity can be absent in moderate forms of optic atrophy, suggesting that future patients with *AFG3L2* mutations should have systematic fundus and OCT examinations. Although the cases we report here do not present the spinocerebellar ataxia typical of SCA28 patients, we cannot rule out the possibility that they will develop this symptom later in their life time.

Finally, both patients showed mild mental retardation, a finding which has not yet been associated to DOA, SCA28, or SPAX5. Whether cognitive disturbances is related to the *AFG3L2* mutation itself, or to an independent phenomenon related to another dominant locus segregating separately from *AFG3L2* was not determined. Therefore, a fortuitous association of DOA and mental deficiency in these patients cannot be ruled out. Nevertheless, we suggest that genetically unresolved DOA patients with or without intellectual disability could be screened for the presence of an *AFG3L2* mutation. In this respect, finding novel *AFG3L2* mutations related to DOA or disclosing optic atrophy in SCA28 patients would strongly reinforce the data that we presented here.

## Concluding Remarks

Dominant Optic Atrophy is among the most frequent mitochondrial disorder and probably the most frequent form of inherited optic neuropathy (Lenaers et al., 2012) and essentially associated to an *OPA1* mutation. Nevertheless exonic sequencing of this gene and *OPA3* and *SPG7*, the other known DOA genes, only leads to some 50% of positive results. Therefore, identification of additional genes involved in DOA is required to help genetic diagnosis. In this respect, the identification of a dominant mutation in *SPG7* and eventually in *AFG3L2* in DOA patients suggests that *m-*AAA proteases could be involved in a pathway responsible of retinal ganglion neuron degeneration, and contribute to a better understanding of the etiology and genotype – phenotype correlations of inherited diseases affecting the optic nerve. Although probably very uncommon, the possible involvement of *AFG3L2* and *SPG7* in optic neuropathies should prompt the screening of these genes in isolated and syndromic DOA patients, negative for *OPA1* and *OPA3* mutation. They also should favor neuro-ophthalmological examinations in SCA28 and SPAX5 patients with spinocerebellar ataxia.

## Author Contributions

All authors contributed to the conception and the design of the study, to the analysis of data, to the writing and approval of the manuscript to be published, and agree to be accountable for all the aspects of the work. AR, SM, CG, and CPH contributed to the clinical investigations. MJ, SS, and GL contributed to the genetic analysis.

## Ethics

Informed written consent was obtained from all patients to perform genetic studies.

The Department of Ophthalmology of the University-Hospital of Montpellier has the authorization # 11018S from the French Ministry of Health to perform biomedical research in the field of physiology, pathophysiology, epidemiology, and genetics in ophthalmology.

## Conflict of Interest Statement

The authors declare that the research was conducted in the absence of any commercial or financial relationships that could be construed as a potential conflict of interest.

## Supplementary Material

TABLE S1 **Listing of the 30 damaging single nucleotide polymorphisms identified in the whole exome sequencing of the patient III.4**.
